# Accessory Apical Foramina in Mandibular Second Molars with Mesial and Distal Root Canal Systems: An Ex Vivo Micro-CT Morphometric Study

**DOI:** 10.3390/jcm15197659

**Published:** 2026-10-02

**Authors:** Katia Greco, Riccardo Federico Visconti, Maria Teresa Sberna, Enrico Felice Gherlone, Giuseppe Cantatore

**Affiliations:** Department of Endodontics, IRCCS San Raffaele Scientific Institute, Dental School, Vita-Salute San Raffaele University, 20132 Milan, Italy; katiagreco@libero.it (K.G.); riccardo.visconti@hotmail.it (R.F.V.); sberna.mariateresa@hsr.it (M.T.S.); gherlone.enrico@hsr.it (E.F.G.)

**Keywords:** accessory apical foramina, mandibular second molar, root canal morphology, micro-computed tomography, Vertucci classification, endodontics

## Abstract

**Background:** The apical anatomy of mandibular second molars may include accessory foramina not adequately represented by classifications of the main root canal configuration. This ex vivo micro-computed tomographic study evaluated the frequency, number, and two-dimensional perimeter of accessory apical foramina associated with independent mesial and distal canal systems. **Methods:** Sixty-four extracted mandibular second molars were selected, including teeth with completely separate roots and teeth with partial external root fusion, provided that the mesial and distal canal systems remained independent and terminated in distinct main foramina. Mesial systems were stratified by Vertucci configuration as Type I (*n* = 21), Type II (*n* = 21), and Type IV (*n* = 22), whereas all distal systems were Type I. Specimens were scanned at an isotropic voxel size of 10.98 μm. Accessory apical foramina were defined as openings distinct from the main apical foramen or foramina that were continuous with the canal system and communicated with the external root surface. Mesial and distal systems were compared using paired analyses. **Results:** Accessory apical foramina were detected in 42/64 mesial systems (65.6%) and 21/64 distal systems (32.8%). Their frequency was significantly higher in mesial systems. Overall, 152 accessory foramina were identified in mesial systems and 66 in distal systems, with significantly higher paired counts mesially (*p* = 0.0002). Among the 15 teeth presenting accessory apical foramina in both the mesial and distal canal systems, the mean number of accessory foramina was similar between the two systems, whereas distal accessory foramina had a larger mean two-dimensional perimeter. Mesial Vertucci configuration was not significantly associated with their presence or number. **Conclusions:** Accessory apical foramina occurred frequently, particularly in mesial canal systems. Main canal configuration did not fully represent apical foraminal complexity, emphasizing the importance of cleaning, disinfection, and obturation of the entire apical canal system. Because the sample was deliberately stratified by mesial canal configuration, the reported frequencies characterize the selected sample and should not be interpreted as population prevalence estimates.

## 1. Introduction

Mandibular second molars play an important functional role in mastication and present some of the most variable and complex endodontic anatomies in the mandibular arch [1]. Their root morphology may include completely separate roots, partially or completely fused roots, supernumerary roots, C-shaped configurations, and other anatomical variants. Similarly, the root canal system may range from relatively simple arrangements to highly complex networks. This variability is clinically relevant because failure to recognize the complete anatomy may compromise root canal shaping, cleaning, disinfection, and three-dimensional obturation [2,3].

The classification proposed by Vertucci remains one of the most widely used systems for describing the main root canal configurations of permanent teeth [4]. In a systematic review of the root anatomy and canal configuration of permanent mandibular second molars, Joshi et al. [5] analyzed 37 studies comprising 12,393 teeth and reported marked morphological heterogeneity. The number of canals ranged from one to five, and the observed configurations included various Vertucci types as well as patterns that could not be adequately described by the original classification. In the mesial root, Vertucci Type IV was the most frequent configuration, with an overall prevalence of 37.3%, and two mesial canals were reported in 70.4% of cases [5]. By contrast, the distal root contained a single canal in 77.0% of cases, and Vertucci Type I was the predominant configuration, with a prevalence of 85.2%. These findings indicate that mesial and distal canal systems should not be considered anatomically equivalent: the mesial system is generally characterized by greater canal division, convergence, and interconnection, whereas the distal system more commonly contains a single main canal.

The endodontic complexity of mandibular second molars, however, extends beyond the number and configuration of the main canals. The apical third may contain accessory and lateral canals, intercanal communications, anastomoses, apical deltas, and multiple apical foramina. These minute structures may be difficult or impossible to reach with endodontic instruments and may be incompletely penetrated by irrigating solutions. Consequently, they may harbor residual organic tissue, biofilm, and microorganisms, and may contribute to persistent intraradicular infection or to communication between the root canal system and the periapical tissues [6,7,8]. The limitations of mechanical instrumentation in complex canal systems are supported by the micro-CT findings of Greco et al. [9]. The authors compared the shaping ability of ProTaper Ultimate and BlueShaper in the mesial and distal canals of mandibular second molars. After canal preparation, the mean percentages of untreated canal surface were 59.25 ± 29.95% for ProTaper Ultimate and 54.62 ± 15.69% for BlueShaper, with no significant difference between the systems. In both groups, distal canals showed significantly higher percentages of untreated surface than mesial canals (*p* < 0.05). Although the study did not specifically evaluate accessory apical foramina or separately analyze the apical third, its findings demonstrate that contemporary nickel–titanium instruments may leave a substantial portion of the canal surface unaffected, particularly in anatomically irregular areas.

Cross-sectional CBCT studies conducted in different populations have demonstrated considerable variation in the external root morphology and internal canal configuration of mandibular second molars. In an analysis of 190 teeth, Gomez et al. [10] found two separate roots in 85.5% of mandibular second molars and three root canals in 87.7%. At the whole-tooth level, 10.0% of the teeth had one apical foramen, 75.3% had two, 13.6% had three, and 1.0% had four. Thus, the predominant anatomy consisted of two roots, three canals, and two apical foramina per tooth, although considerable variation was observed. However, the foraminal data were not reported separately for the mesial and distal canal systems, and the study did not distinguish main from accessory apical foramina.

Considerable anatomical variability has also been reported in Asian populations. In a Chinese sample of 1200 mandibular second molars, Yang et al. [11] identified two separate roots in 61.0% of teeth and a C-shaped root in 35.6%. In two-rooted teeth, Vertucci Types II and IV were the most frequent mesial configurations, whereas the distal root displayed a Type I configuration in 95.6% of cases. Using canal staining and tooth clearing, Neelakantan et al. [12] similarly found that two roots and three canals represented the predominant morphology in an Indian population, with Vertucci Type IV and Type I configurations prevailing in the mesial and distal roots, respectively. Approximately 54.8% of the teeth presented two apical foramina. As in the study by Gomez et al. [10], foraminal counts were reported at the whole-tooth level, without distinguishing main from accessory foramina or providing separate morphometric data for mesial and distal canal systems. In a Korean CBCT study, Kim et al. [13] reported one root in 41.0% and two roots in 58.0% of mandibular second molars, with Vertucci Types IV and II predominating in the mesial roots of two-rooted teeth. Pawar et al. [14] also identified two-rooted, three-canal molars as the most common pattern in an Indian population, along with C-shaped systems and less frequent anatomical variants. Martins et al. [15] further demonstrated population-related differences, finding a higher prevalence of single-rooted mandibular second molars in Asian than in White individuals (45.4% versus 14.3%). Conversely, Almansour et al. [16] reported two roots in 94.1% of teeth from a Saudi subpopulation. Using CBCT, Senan et al. [17] also identified canal configurations beyond the classic Vertucci types in mandibular second molars from a Yemeni population, including multiple canals that converged or maintained separate apical pathways.

Collectively, these findings demonstrate marked geographic and ethnic variation in root number, root fusion, canal configuration, and the prevalence of C-shaped systems. However, these investigations primarily focused on root morphology, main canal configurations, and C-shaped anatomy rather than on the distribution and dimensions of accessory apical foramina. Although the total number of apical exits was recorded in some studies [10,12], main and accessory apical foramina were not systematically differentiated. Moreover, accessory foramina were not separately quantified for mesial and distal canal systems, and their dimensions were not measured. This lack of data is partly methodological. Clinical CBCT is suitable for identifying major variations in root morphology and main canal configuration in vivo; however, its spatial resolution and susceptibility to partial-volume effects may limit the reproducible detection and measurement of minute ramifications and root-surface openings. Consequently, CBCT generally does not allow accessory apical foramina to be characterized morphometrically at the level of detail achievable with high-resolution micro-CT.

Micro-computed tomography enables nondestructive, three-dimensional evaluation of internal and external dental anatomy at substantially higher spatial resolution. Wolf et al. [18] demonstrated its ability to characterize the geometry and dimensions of physiological foramina in molars, whereas Xu et al. [19] used this technique to assess the diameter, length, shape, and course of apical accessory canals. Using micro-CT, Cheung et al. [20] demonstrated that mandibular second molars with C-shaped canal systems have particularly complex apical anatomy, characterized by accessory and lateral canals, anastomoses, and apical deltas. Approximately 80% of the examined systems exhibited one to three apical foramina, and accessory foramina were observed in approximately 48% of cases. Collectively, these studies demonstrate that micro-CT permits detailed reconstruction of the root canal system and its minute apical ramifications while preserving the original specimen, thereby overcoming several limitations of conventional two-dimensional methods and clinical CBCT. They also show that the number of main canals does not necessarily correspond to the number of root-surface exits.

Despite these advances, quantitative micro-CT data on accessory apical foramina in non-C-shaped mandibular second molars remain limited. Specifically, it remains unclear whether their occurrence and morphometric characteristics differ between anatomically independent mesial and distal canal systems and whether their presence in the mesial system is associated with the Vertucci type. Importantly, separate evaluation of the two canal systems is warranted because their anatomical differences may be linked to distinct patterns of apical ramification. Therefore, the aim of this ex vivo micro-CT study was to evaluate the frequency, number, and two-dimensional perimeter of accessory apical foramina in anatomically independent mesial and distal canal systems of mandibular second molars. Main apical foramina served as anatomical reference structures for distinguishing them from accessory openings and were not considered independent quantitative endpoints. The null hypotheses were as follows:

(1) There are no significant differences in the frequency, number, or two-dimensional perimeter of accessory apical foramina between anatomically independent mesial and distal canal systems of mandibular second molars.
(2) The presence of accessory apical foramina in the mesial canal system is not associated with the mesial Vertucci type.

## 2. Materials and Methods

### 2.1. Study Design and Specimen Selection

This ex vivo study analyzed 64 two-rooted mandibular second molars, each obtained from a different Caucasian patient aged 55–60 years and extracted for periodontal reasons.

**Figure 1:**
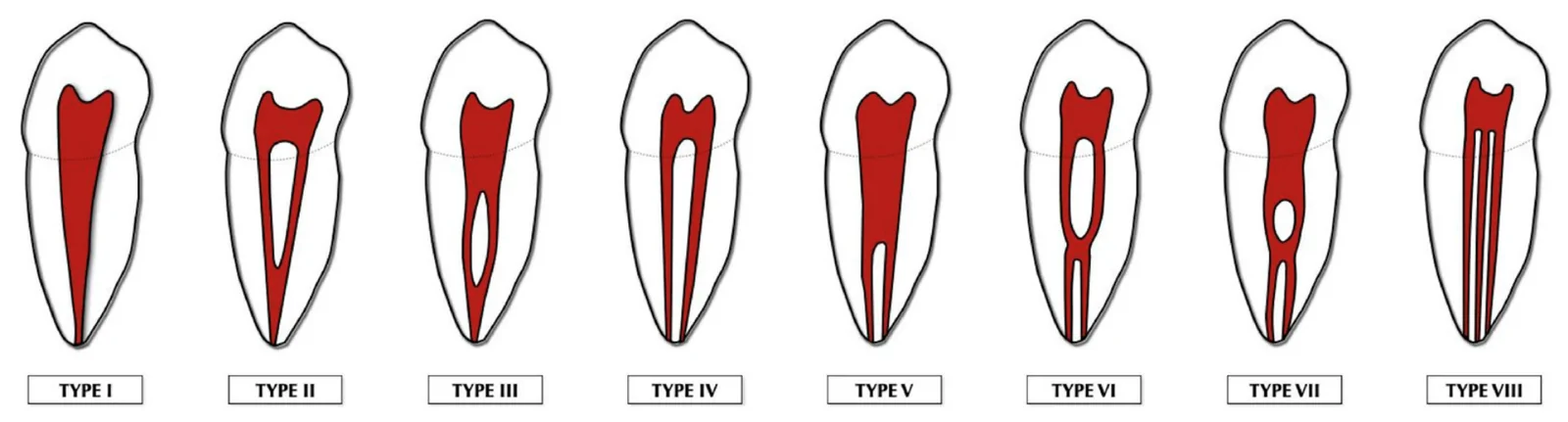
Schematic representation of the Vertucci root canal configuration classification (Types I–VIII). Each panel shows the canonical pattern of canal splitting and merging from the pulp chamber to the apical foramen.

Specimens were selected using non-probability stratified sampling to ensure balanced representation of the three mesial root canal configurations according to Vertucci’s classification (Figure 1). From a larger collection of extracted molars, specimens were assigned to three predefined groups based on mesial canal configuration until approximately equal group sizes were achieved: Type I (*n* = 21), Type II (*n* = 21), and Type IV (*n* = 22). In all selected specimens, the distal canal system exhibited a Type I configuration. Because of this stratified selection, the observed frequencies do not represent natural prevalence estimates of canal configurations or apical foramina in the general population. Instead, they describe the morphometric characteristics of the specific configurations under study.

The inclusion criteria required mandibular second molars with anatomically independent mesial and distal canal systems that terminated in distinct main apical foramina. Teeth with macroscopically separate roots and those with partial external root fusion in the middle or apical third were included, provided that canal-system independence was fully preserved. All teeth had fully formed apices.

Exclusion criteria included single-canal or C-shaped configurations, previous endodontic treatment, extensive carious destruction, root fracture, severe resorption, marked hypercementosis, or any other structural alteration that could compromise the analysis of apical anatomy. Because the teeth had been extracted from adult patients for periodontal reasons, the integrity of the apical 3 mm was verified on reconstructed micro-CT images rather than by macroscopic inspection alone. Specimens showing alterations in the apical region were excluded to minimize potential anatomical bias associated with age or the reason for extraction. After extraction, the teeth were gently cleaned of soft-tissue residues and hard deposits on the root surface, avoiding instrumentation near the apex that could alter the foramen openings. Samples were stored in saline at room temperature and scanned by micro-CT within 10 days. Each sample was initially radiographed in two projections, mesiodistal and buccolingual, to assess root and canal morphology. Canal configuration was then classified according to Vertucci based on reconstructed micro-CT images.

The mesial and distal root canal systems were considered the primary anatomical units of analysis. Because the two systems originated from the same tooth, their paired structure was explicitly accounted for in the statistical analysis.

### 2.2. Operational Definition of Main and Accessory Apical Foramina

Predefined operational definitions were adopted and uniformly applied to all specimens to avoid terminological ambiguity between the terminal exits of the main canals and accessory openings.

The main apical foramen was defined as the terminal exit of a main root canal in direct continuity with the canal lumen [21]. In root canal systems with two independent main canals that reached the apical surface separately, as in Vertucci Type IV configurations, both terminal exits were classified as main foramina and were not counted as accessory foramina. Identification of main foramina was performed solely to distinguish the expected anatomical exits of the main canals from accessory openings; the number of main foramina was not treated as a separate quantitative endpoint of the study.

An accessory apical foramen was defined as any additional opening, distinct from the main apical foramen or foramina, located in the apical region, anatomically continuous with the root canal system, and communicating with the external root surface [22]. A root canal system was classified as positive if it had at least one accessory apical foramen in addition to the main apical foramen or foramina.

For each system, the recorded variables were the presence or absence of accessory apical foramina, their total number, the perimeter of each opening, and, in positive systems, the mean perimeter at the system level.

### 2.3. Study Endpoints and Sample Size

The primary endpoint was the paired difference in the presence of at least one accessory apical foramen between the mesial and distal root canal systems of the same tooth. Secondary endpoints included the count of accessory apical foramina per system and the average two-dimensional perimeter of accessory foramina in systems containing at least one accessory apical foramen.

To determine the required sample size, an a priori power analysis was conducted in G*Power (version 3.1.9.7; Heinrich Heine University Düsseldorf, Düsseldorf, Germany) [23,24]. The primary dichotomous endpoint (presence or absence of accessory apical foramina) was analyzed using McNemar’s test for paired proportions, with α = 0.05, 90% power (1 − β = 0.90), and an expected odds ratio of 3.0. The analysis indicated that at least 30 paired observations were required. In the present study, 64 teeth, providing 64 paired observations (one mesial and one distal canal system per tooth), were analyzed, exceeding the minimum sample size required for the primary endpoint.

### 2.4. Micro-CT Acquisition

All specimens were scanned using a SkyScan 1172 micro-computed tomography scanner (Bruker microCT, Kontich, Belgium), equipped with an 11-megapixel camera. Each tooth was positioned individually along the acquisition axis and mechanically stabilized to prevent motion artifacts. The acquisition parameters were kept constant for all specimens: isotropic voxel size, 10.98 µm; source voltage, 70 kV; current, 141 µA; 0.5 mm aluminum filter; exposure time, 850 ms; frame averaging, 4; angular rotation step, 0.45°; and rotation arc, 180°. A total of 400 projections were acquired for each specimen.

### 2.5. Image Reconstruction and Dataset Orientation

The acquired radiographic projections were reconstructed using NRecon software (version 1.6.9.8; Bruker microCT, Kontich, Belgium), with the Feldkamp cone-beam reconstruction algorithm [25]. Reconstruction generated stacks of two-dimensional axial sections perpendicular to the acquisition axis, with an interslice step of 1 voxel, corresponding to 10.98 µm. Thus, approximately 91 sections were obtained for each millimeter of specimen height. The reconstruction settings included an angular step of 0.45°, ring-artifact correction of 9, and beam-hardening correction of 20%. The reconstructed datasets were standardized using DataViewer software (version 1.5.6.2; Bruker microCT, Kontich, Belgium), with the long axis of each canal system oriented as nearly parallel as possible to the analytical Z-axis. This orientation facilitated reproducible assessment of the apical region and comparison of mesial and distal systems.

For each system, an apical reference section was identified as the section in which the main canal lumen communicated with the external root surface and was no longer completely surrounded by dentin along its perimeter. This section was designated as slice 0 for anatomical tracking of the apical canal system. From slice 0, levels located 1, 2, 3, 4, and 5 mm coronally were identified by adding approximately 91, 182, 273, 364, and 455 sections, respectively, to the reference section number. These levels supported evaluation of the anatomical continuity and course of the main and accessory canals in the apical region but were not considered independent quantitative endpoints of the present study.

### 2.6. Identification of Accessory Foraminal Openings

The apical volume of each system was examined section by section along the Z-axis. The analysis combined evaluation of two-dimensional axial sections with inspection of adjacent sections and three-dimensional reconstructions to distinguish true accessory foraminal openings from surface irregularities, isolated porosities, mineralization defects, microcracks, or segmentation artifacts. Each accessory foraminal opening was identified using a specific foraminal reference section, defined as the section in which the segmented lumen communicated with the external root surface and the contour of the opening was clearly delineated. Adjacent coronal and apical sections were used to confirm anatomical continuity of the opening with the root canal system.

When an accessory canal followed an oblique course, the perimeter was measured on the section in which the opening was most clearly identifiable as a communication between the canal lumen and the external root surface. Therefore, the perimeter of each accessory foramen was measured on its own foraminal reference section and not on a single common section for the entire root. This approach allowed inclusion of accessory openings located on slightly different axial planes and reduced the risk of overlooking oblique, eccentric, or apical openings located on root surfaces not perfectly perpendicular to the acquisition axis.

### 2.7. Segmentation and Morphometric Analysis

Selected sections were imported into CT Analyzer (CTAn, version 1.20.8.0; Bruker microCT, Kontich, Belgium) and processed according to a standardized workflow. Grayscale images were binarized using Otsu automatic thresholding [26], separating the hard root tissue from the void spaces corresponding to canal lumens and foraminal openings. The threshold was applied individually to each image, and segmentation accuracy was visually verified on adjacent sections and through three-dimensional reconstructions.

**Figure 2:**
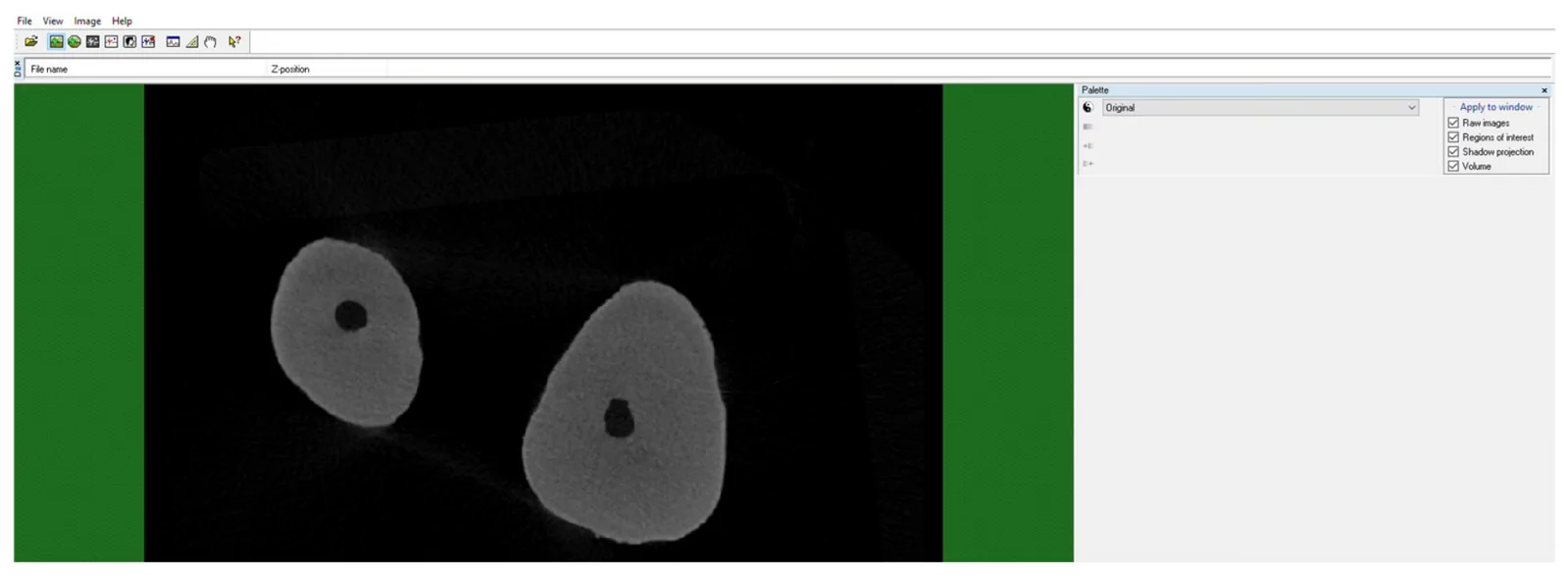
Example of an axial micro-CT section of the apical region of a mandibular second molar with independent mesial and distal root canal systems.

**Figure 3:**
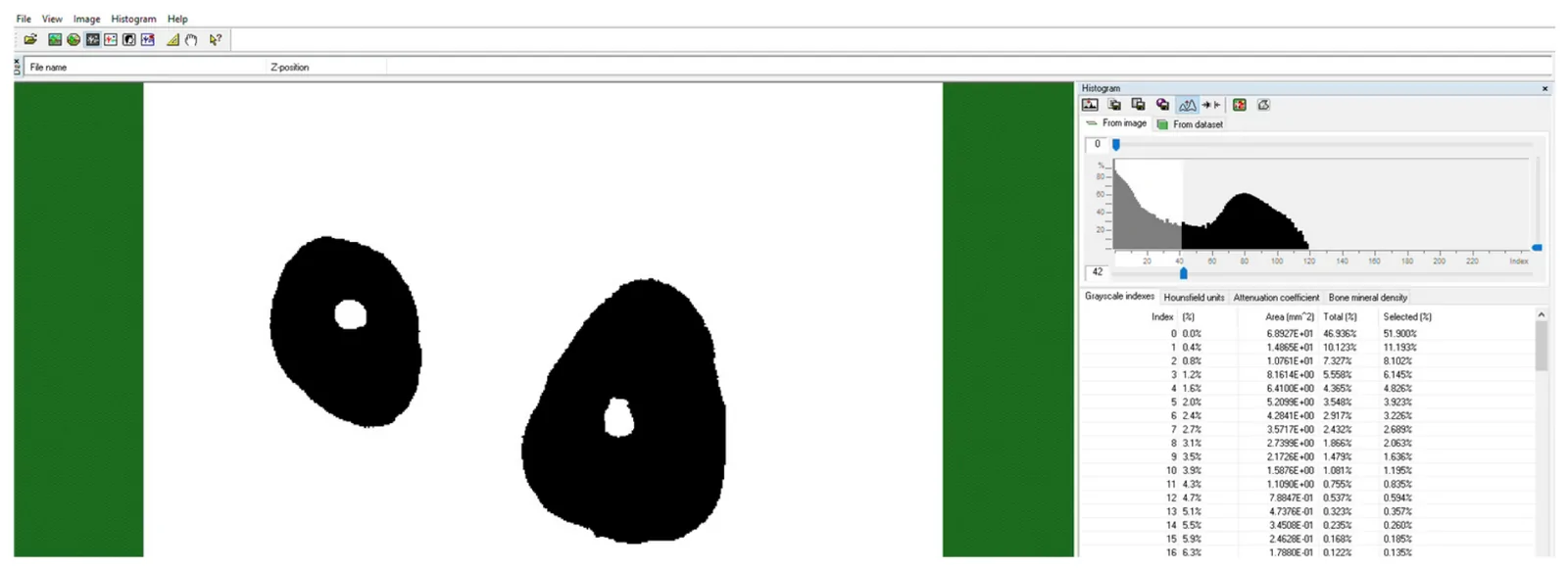
Example of binary segmentation of root canals and foraminal openings using CT Analyzer.

**Figure 4:**
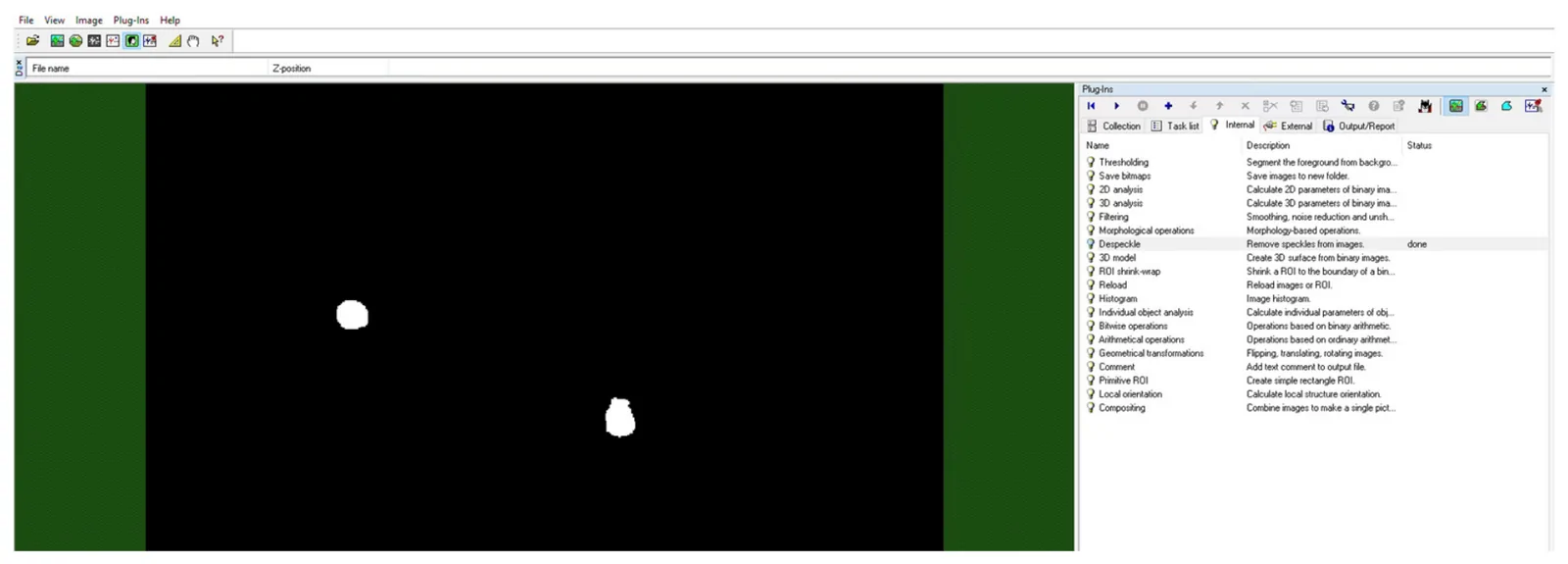
Isolation of segmented objects corresponding to the foraminal openings in the reference section.

**Figure 5:**
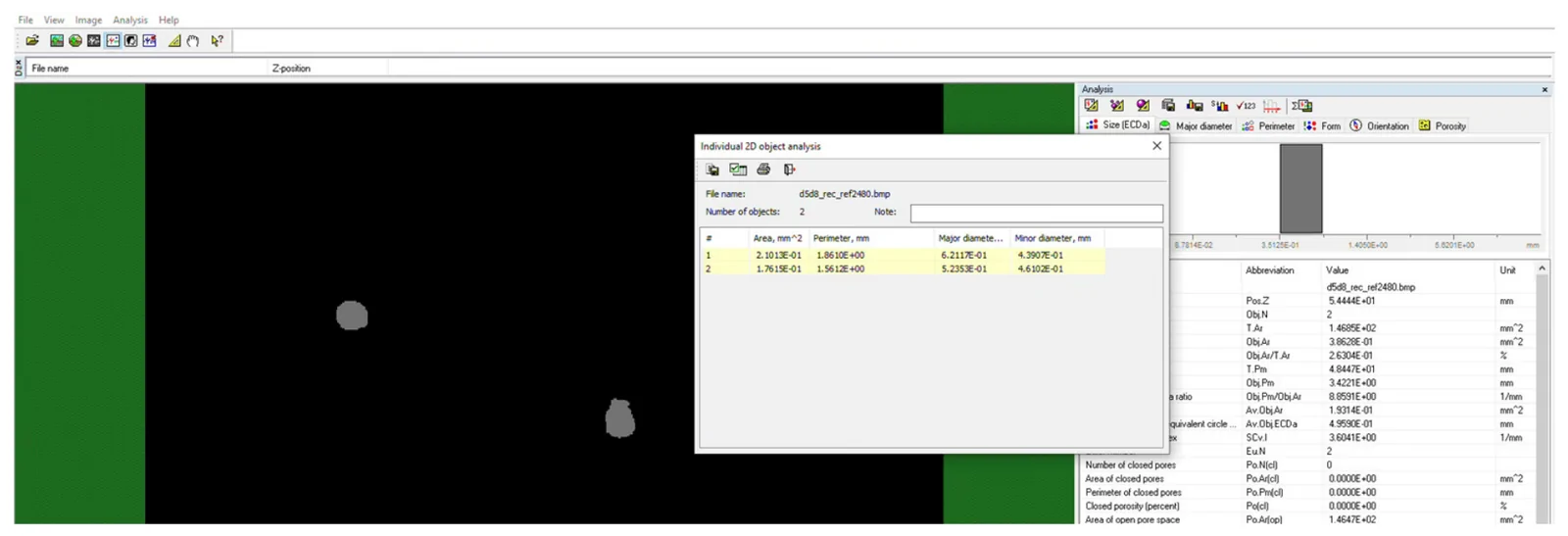
Example of individual object analysis in CT Analyzer with calculation of the two-dimensional perimeter of accessory foramina. The CTAn output window also displays automatically generated area and major/minor diameter values; only perimeter was a predefined endpoint and was systematically retained for all accessory openings.

After binarization, the binary image was inverted so that the endodontic spaces—including the main canal lumen, accessory canals, and foraminal openings—were represented as foreground objects. The external background was then removed, leaving only objects attributable to the root canal system. Each connected group of contiguous foreground pixels was treated as an individual object for two-dimensional morphometric analysis. The workflow, from axial micro-CT imaging and binary segmentation to object isolation and morphometric analysis, is illustrated in Figure 2, Figure 3, Figure 4 and Figure 5.

A structure was considered an accessory apical foramen only if it met the following criteria simultaneously:

- Anatomical continuity with the canal system;
- Direct communication with the external root surface;
- Localization within the apical region;
- Visibility across consecutive sections or confirmation via 3D reconstruction;
- Minimum size of at least three contiguous voxels along the minor diameter of the segmented object (corresponding to approximately 33 µm at the current resolution);
- Absence of morphological features suggestive of an isolated defect, surface porosity, microcrack, artifact, or segmentation noise.

A minimum-dimensional threshold of at least three times the voxel size was adopted as a conservative criterion to reduce the risk of classifying structures represented by only a few isolated pixels as accessory foramina, which are particularly sensitive to the partial-volume effect and to threshold selection. The three-voxel criterion was used solely as a minimum-dimensional eligibility threshold; the corresponding minor-diameter values were not retained as morphometric outcomes.

The perimeter of each accessory foramen was calculated in CT Analyzer as the length of the two-dimensional contour of the segmented object on the foraminal reference section, using the Pratt algorithm [27]. Values were calibrated to the voxel size and expressed in millimeters. This measurement reflects the 2D contour of the opening within the selected section rather than the absolute 3D perimeter on the root surface. Although the software automatically generates additional morphometric parameters (e.g., area, major diameter, and minor diameter), this study’s quantitative analysis was limited to the predefined endpoints: presence, number, and perimeter of accessory apical foramina.

Perimeter was selected as a predefined secondary morphometric endpoint because it directly characterizes the boundary length of the segmented opening and can be applied to irregular, non-circular contours without assuming a circular shape. It was intended as a comparative boundary-length descriptor sensitive to both opening dimensions and contour irregularity, rather than as a measure of complete three-dimensional geometry or as a surrogate for fluid exchange or clinical risk. Area and major/minor diameters could in principle be generated from the same two-dimensional segmentation; however, they were not prespecified or systematically recorded in the retained morphometric dataset and were therefore not introduced as post hoc outcomes. Equivalent diameter summarizes the area of an opening as the diameter of a circle with the same area and may therefore obscure irregularity of the foraminal contour; it would provide complementary rather than interchangeable information. Three-dimensional surface descriptors would require a separate surface-based segmentation and orientation-normalization protocol.

To prevent pseudoreplication, individual foramina were not treated as independent statistical units when multiple accessory openings occurred within the same canal system. Instead, the mean system-level perimeter was calculated, yielding one observation per positive system for the inferential morphometric analyses [28].

### 2.8. Statistical Analysis

Statistical analyses were conducted using IBM SPSS Statistics (version 30.0; IBM Corp., Armonk, NY, USA). Additional analyses conducted during revision used Python 3.12, SciPy 1.18.1, and statsmodels 0.15.0. The significance level was set at *p* < 0.05. Categorical variables were reported as absolute frequencies and percentages; continuous variables were reported as mean ± standard deviation, median, interquartile range, and range, according to their distribution. Normality of continuous variables was assessed using the Shapiro–Wilk test [29].

The primary endpoint, defined as the presence of at least one accessory apical foramen in the mesial root canal system compared with the distal system of the same tooth, was analyzed as paired data using the McNemar exact test [30]. The matched odds ratio was calculated from discordant pairs, and a 95% confidence interval was estimated using an exact method based on the binomial distribution of discordant pairs.

The number of accessory foramina per canal system was analyzed as a count variable. Given the paired data structure and the expected non-normal distribution of the counts, mesial and distal canal systems were compared using the Wilcoxon signed-rank test [31]. Mean counts were reported for all systems and for positive systems only.

As a supplementary analysis conducted during revision, the number of accessory foramina was modeled using generalized estimating equations (GEEs) [32], with a log link, a Poisson working variance, canal-system location (mesial or distal) as the predictor, and tooth as the clustering unit. All 128 systems from 64 teeth were included, with zero counts retained. An independence working correlation structure was used together with the Mancl–DeRouen bias-reduced robust sandwich covariance estimator [33], allowing inference to account for within-tooth dependence and departures from the Poisson variance assumption. Results were expressed as the mesial-to-distal mean count ratio with a 95% confidence interval. Wald confidence intervals and two-sided *p*-values used the normal approximation. A negative binomial model with a tooth-specific normally distributed random intercept was also evaluated by maximum likelihood using 80-point Gauss–Hermite quadrature; its estimated random-intercept variance was zero. The GEE models included canal-system location only and did not adjust for Vertucci configuration.

The perimeter analysis included only systems containing at least one accessory foramen. Mean system-level perimeter was compared between mesial and distal systems using a Gaussian identity-link GEE, with canal-system location as the predictor and tooth as the clustering unit [32]. An independence working correlation structure and the Mancl–DeRouen bias-reduced robust sandwich covariance estimator [33] were used to account for within-tooth dependence and allow unequal variability between mesial and distal systems. Each positive system contributed one mean perimeter value, regardless of the number of accessory openings. The analysis included 63 positive systems from 48 teeth and estimated the distal–mesial difference with a 95% confidence interval. Wald confidence intervals and two-sided *p*-values used the normal approximation. Systems without accessory foramina had no defined perimeter and were excluded from this analysis rather than assigned a value of zero. As a sensitivity analysis, mean perimeter values were compared using the paired-samples *t*-test and Wilcoxon signed-rank test in the 15 teeth with accessory foramina in both systems. All perimeter comparisons were conditional on the presence of accessory foramina.

The association between mesial canal configuration (Vertucci Types I, II, and IV) and the presence of accessory foramina was evaluated using either the Pearson chi-square test or Fisher’s exact test, as appropriate [34,35]. The strength of association was expressed using Cramer’s V. The number of accessory foramina across the three Vertucci groups was compared using the Kruskal–Wallis test [36]; if significant, post hoc comparisons with Holm–Bonferroni correction were planned [37].

### 2.9. Measurement Reproducibility

Image reconstruction and perimeter measurements were performed by an endodontist experienced in imaging software. A second experienced endodontist verified the images, the identification of accessory foraminal openings, and the delineation of their contours. The assessment followed the predefined operational criteria reported in Section 2.6 and Section 2.7, including anatomical continuity with the root canal system, direct communication with the external root surface, confirmation on consecutive sections or three-dimensional reconstructions, the minimum three-voxel dimensional threshold, and exclusion of porosities, microcracks, and segmentation artifacts. Uncertain findings were jointly reassessed using adjacent sections and three-dimensional reconstructions until consensus was reached. The consolidated measurements were used in the final statistical analyses. Separate pre-consensus assessments were not retained; therefore, quantitative interobserver agreement could not be calculated retrospectively.

### 2.10. Ethical Aspects

The study was conducted exclusively on human teeth extracted for clinical reasons unrelated to the research. All specimens were fully anonymized before analysis, and no patient-identifying or clinical information was collected. In accordance with applicable institutional requirements, formal approval by the local ethics committee was not required. The study was conducted in accordance with the relevant principles of the Declaration of Helsinki [38].

## 3. Results

### 3.1. Frequency of Accessory Apical Foramina

At least one accessory apical foramen was observed in 42/64 mesial root canal systems (65.6%) and in 21/64 distal canal systems (32.8%). The distribution of mesial Vertucci configurations and the presence of accessory apical foramina are reported in Table 1.

Because the mesial and distal canal systems originated from the same 64 teeth, the comparison was performed using a paired analysis. The distribution of pairs was as follows: 15 teeth exhibited accessory foramina in both systems, 27 were positive only in the mesial system, 6 were positive only in the distal system, and 16 were negative in both systems (Table 2). The exact McNemar test showed a statistically significant difference in the frequency of accessory apical foramina between the mesial and distal canal systems (*p* = 0.0003). The matched odds ratio (OR), calculated from the discordant pairs, was 4.5 (95% confidence interval [CI]: 1.82–13.33). Thus, within the analyzed sample, mesial systems exhibited a significantly higher frequency of accessory apical foramina than the corresponding distal systems of the same tooth.

### 3.2. Number of Accessory Apical Foramina per System

A total of 152 accessory apical foramina were identified in the mesial canal systems and 66 in the distal canal systems. When all 64 mesial–distal canal-system pairs were considered, the mean number of accessory foramina was 2.38 per mesial system and 1.03 per distal system. The Wilcoxon signed-rank test showed a significantly higher count in mesial systems (*p* = 0.0002). The supplementary GEE analysis accounting for tooth-level clustering yielded a mesial-to-distal mean count ratio of 2.30 (95% CI, 1.48–3.58; *p* = 0.000207), consistent with the paired comparison. Among positive systems (containing at least one accessory foramen), the corresponding mean counts were 3.62 mesially and 3.14 distally. Descriptive data on the total and mean numbers of accessory apical foramina are presented in Table 3.

**Figure 6:**
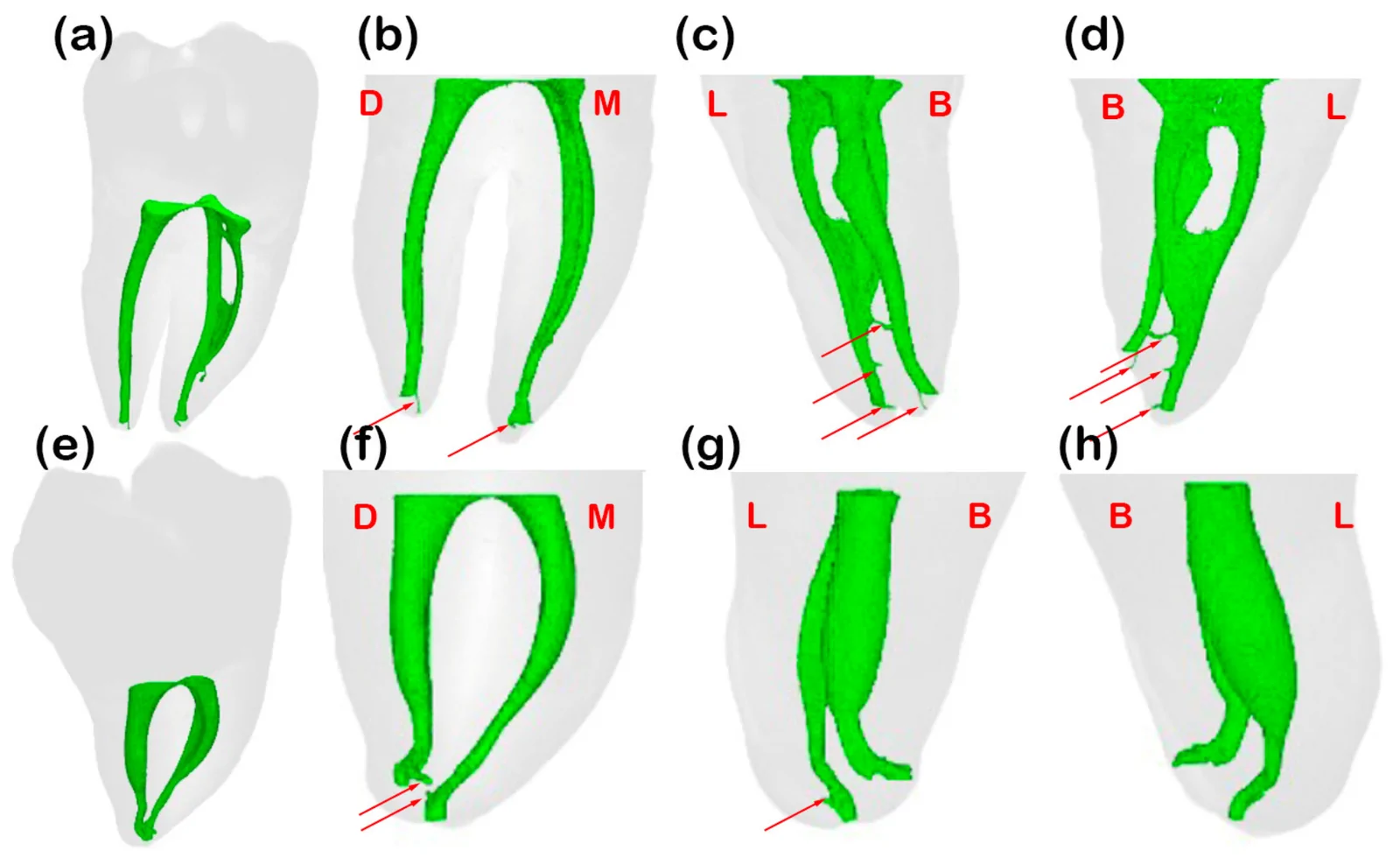
Representative micro-CT three-dimensional reconstructions of mandibular second molars with anatomically independent mesial and distal canal systems. (**a**–**d**) Tooth 1, exhibiting macroscopically separate roots: (**a**) three-dimensional axonometric reconstruction showing the overall root canal anatomy; (**b**) sagittal view; (**c**) mesial coronal view; and (**d**) distal coronal view. (**e**–**h**) Tooth 2, exhibiting partial external root fusion beginning in the middle third, while maintaining anatomically independent mesial and distal canal systems that terminate in distinct main apical foramina: (**e**) three-dimensional axonometric reconstruction; (**f**) sagittal view; (**g**) mesial coronal view; and (**h**) distal coronal view. Red arrows indicate accessory apical foramina. M, mesial; D, distal; B, buccal; L, lingual.

**Figure 7:**
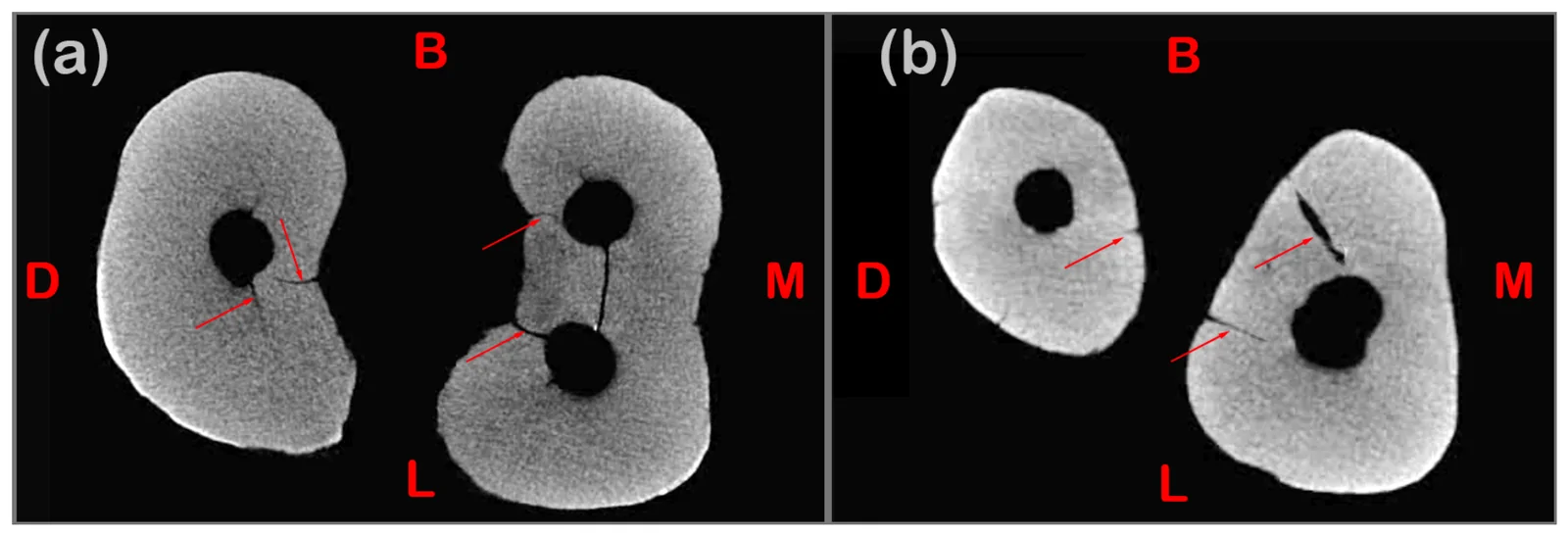
Representative axial micro-CT sections from the reconstructed image stacks of the same mandibular second molars shown in Figure 6, selected at apical levels. (**a**) Tooth 1, exhibiting macroscopically separate mesial and distal roots with anatomically independent canal systems. (**b**) Tooth 2, exhibiting partial external root fusion on three-dimensional reconstruction, while maintaining anatomically independent mesial and distal canal systems. The axial sections show accessory apical ramifications corresponding to the accessory foramina indicated in Figure 6. Red arrows indicate the accessory ramifications. M, mesial; D, distal; B, buccal; L, lingual.

Morphological assessment revealed substantial variability in apical anatomy. The visibility of accessory ramifications varied by viewing projection: in the sagittal plane, they were partially or completely obscured by the overlying root structure, whereas the mesial and distal coronal views allowed clearer identification. The three-dimensional distribution of accessory apical foramina is illustrated in Figure 6, while the corresponding axial micro-CT sections in Figure 7 show the associated apical ramifications and their relationship to the mesial and distal canal systems.

### 3.3. Accessory Apical Foramina and Vertucci Configuration in Mesial Systems

Mesial canal systems were classified as Vertucci Type I (*n* = 21), Type II (*n* = 21), or Type IV (*n* = 22). Positivity rates were 13/21 (61.9%), 15/21 (71.4%), and 14/22 (63.6%), respectively. Pearson’s chi-square test showed no significant association between canal configuration and the presence of accessory apical foramina (χ^2^ = 0.48, df = 2, Cramer’s V = 0.087, *p* = 0.786). Similarly, the number of accessory apical foramina did not differ significantly among the three configurations (Kruskal–Wallis H = 1.10, df = 2, *p* = 0.577). No statistically significant differences in the presence or number of accessory apical foramina were detected among the three mesial Vertucci groups. These within-mesial comparisons do not establish whether the overall mesial–distal difference is independent of canal configuration. The distribution of positive mesial systems and the corresponding overall comparisons are reported in Table 4.

### 3.4. Perimeter of Accessory Apical Foramina

The perimeter of accessory apical foramina was analyzed at the system level by calculating the mean perimeter of openings in each positive root canal system. The analysis included 42 positive mesial systems and 21 positive distal systems. This comparison was conditional on the presence of at least one accessory foramen. The mean system-level perimeter was 0.172 ± 0.028 mm in mesial systems and 0.432 ± 0.176 mm in distal systems. The corresponding medians were 0.168 mm (IQR, 0.151–0.190; range, 0.114–0.236) and 0.395 mm (IQR, 0.302–0.558; range, 0.186–0.780), respectively (Table 5).

In the GEE analysis of the 63 positive canal systems from 48 teeth, mean system-level perimeter was greater in distal than in mesial systems. The estimated distal–mesial difference was 0.260 mm (95% CI, 0.184–0.337; *p* < 0.001), with uncertainty estimated using bias-reduced robust sandwich standard errors clustered by tooth (Table 6). The sensitivity analysis restricted to the 15 teeth with accessory apical foramina in both canal systems yielded consistent results in the paired comparisons (paired *t*-test: t = 6.10, df = 14, *p* < 0.001; Wilcoxon signed-rank test: *p* < 0.001).

## 4. Discussion

This micro-computed tomography (micro-CT) study analyzed the frequency, number, and two-dimensional perimeter of accessory apical foramina associated with anatomically independent mesial and distal canal systems in mandibular second molars. Accessory foramina were present in 65.6% of mesial systems and 32.8% of distal systems. McNemar’s exact test confirmed a significant paired difference, with a matched odds ratio of 4.5, indicating a higher frequency in mesial systems in the selected sample.

The greater frequency observed in mesial systems may reflect anatomical differences in apical ramification between mesial and distal systems; however, the present study does not establish the anatomical mechanism underlying this difference.

Manning [1] documented marked variability in the root canal anatomy of mandibular second molars, reporting lateral canals in 72% of teeth, predominantly in the apical third, and observing that the apical foramen coincided with the anatomical apex in only 33% of cases. Similarly, Joshi et al. [5] found that Vertucci Type IV was the most prevalent mesial configuration (37.3%) and that two canals were present in 70.4% of mesial roots. In contrast, a single canal was present in 77.0% of distal roots, in which Type I was the predominant configuration (85.2%).

Evidence from in vivo cone-beam computed tomography studies further supports distinct mesial and distal anatomical patterns. Gomez et al. [10], for example, reported a predominance of Vertucci Type II configurations in mesial systems (74.3%) and Type I configurations in distal systems (97.2%). Comparable mesial–distal differences have been documented in Chinese, Indian, and Saudi populations, with Vertucci Types II or IV generally predominating mesially and Type I predominating distally [11,14,16].

However, findings from these studies are not directly comparable with the present results because in vivo CBCT investigations primarily characterize root morphology and main canal configurations, whereas the present micro-CT study specifically evaluated accessory openings distinct from the main apical foramina. A key methodological feature was therefore the operational distinction between main and accessory apical foramina. The expected terminal exits of the main canals—including the two independent exits typically present in mesial systems with a Vertucci Type IV configuration—were excluded from the accessory-foramen count. Consequently, the analysis was restricted to additional openings of the apical canal system potentially associated with lateral canals, secondary canals, apical ramifications, or apical deltas [21,22].

The apical complexity of mandibular second molars is well documented in teeth with C-shaped configurations. Manning [39] reported that among 19 C-shaped mandibular second molars, lateral canals were present in all roots, transverse anastomoses in 15 roots, and apical deltas in 16 roots. Subsequent micro-CT investigations, including that of Fan et al. [40], confirmed the marked three-dimensional complexity of C-shaped root canal systems. Cheung et al. [20] identified accessory canals in 41.0%, lateral canals in 25.0%, intercanal communications in 27.0%, and apical deltas in 11.0% of C-shaped systems. Approximately 80.0% of those configurations possessed one to three apical foramina, and accessory foramina were present in roughly 48.0%.

The present study differs from those investigations by focusing exclusively on mandibular second molars with anatomically independent mesial and distal canal systems, including teeth with separate roots and those with partial external fusion limited to the middle or apical third. C-shaped systems were excluded because their anatomy would prevent reliable assignment of accessory openings to a mesial or distal canal system. The findings therefore demonstrate that substantial apical complexity is not confined to C-shaped anatomy and may also occur when the internal mesial and distal systems remain distinct despite partial external root fusion.

Mesial accessory apical foramina were also analyzed in relation to the Vertucci canal configuration. Although Type II systems had the highest proportion of cases with accessory apical foramina (71.4%, compared with 61.9% for Type I and 63.6% for Type IV), neither their presence nor their number differed significantly among the three configuration groups. This comparison addresses variation within mesial systems and is distinct from the paired mesial–distal comparison. Vertucci classification describes the configuration of the main canals [4], whereas the present outcome concerns additional apical openings distinct from their main terminal exits. Accessory foramina were observed in all three mesial configurations, including Type I, and the two main exits of Type IV systems were excluded from accessory-foramen counts. The higher mesial frequency may therefore involve features of apical ramification not represented by main canal configuration alone, but this explanation remains a hypothesis.

The nonsignificant findings among mesial Vertucci groups should not be interpreted as evidence of equivalence or absence of an association, particularly given the limited group sizes of 21–22 specimens. Moreover, all distal systems were Type I, whereas mesial systems were deliberately selected to represent Types I, II, and IV. The overall mesial–distal comparison therefore does not isolate an effect of canal-system location independent of configuration. The supplementary GEE analyses account for within-tooth clustering but do not adjust for Vertucci configuration. Consequently, the present results establish a difference within the selected sample but do not determine its anatomical mechanism.

The total number of accessory apical foramina was significantly higher in mesial than in distal systems. Among positive systems, however, the mean numbers of accessory apical foramina were similar, with values of 3.62 mesially and 3.14 distally. The higher overall mesial count therefore appears to reflect mainly the greater proportion of positive mesial systems rather than a markedly greater number of accessory openings within each positive system.

The selected morphometric parameter was the two-dimensional perimeter of the segmented foraminal contour. Among positive canal systems, distal accessory apical foramina had a greater mean system-level perimeter than their mesial counterparts. Because this was a secondary analysis restricted to positive systems, the finding should be interpreted cautiously. Because perimeter is influenced by both opening dimensions and contour irregularity, a larger value should be interpreted only as a difference in the measured two-dimensional boundary. It does not necessarily indicate a proportionally larger area, a larger true three-dimensional exit surface, or greater clinical relevance; area, equivalent diameter, major/minor diameters, and three-dimensional descriptors would provide complementary rather than interchangeable information.

Direct comparison with previous morphometric studies is limited because most focused on the diameter, shape, or location of the main apical foramen rather than the perimeter of accessory openings. Wolf et al. [18] used micro-CT to demonstrate substantial variability in the three-dimensional geometry of physiological foramina in molars. Briseño-Marroquín et al. [41] likewise described variations in shape and dimensions, whereas Morfis et al. [42] reported marked apical heterogeneity using scanning electron microscopy. Collectively, these studies confirm the morphological variability of the apical region but do not provide directly comparable numerical data on the two-dimensional perimeter of accessory apical openings.

Accordingly, the first null hypothesis was rejected because mesial and distal canal systems differed significantly in the frequency and number of accessory apical foramina and, among positive systems, in their mean system-level perimeter. The second null hypothesis was not rejected because the presence of accessory apical foramina was not significantly associated with the mesial Vertucci type; the corresponding count comparison was also nonsignificant.

Accessory openings establish anatomical connections between the root canal system and the periapical tissues. De-Deus [43] documented the frequent occurrence of lateral, secondary, and accessory canals, particularly in the apical third. These ramifications may harbor residual tissue, microorganisms, and biofilms that are difficult to reach during treatment. Persistent intraradicular infection is a recognized contributor to post-treatment apical periodontitis [6,7,8,44], whereas tissues within lateral canals and apical ramifications may respond differently to disease and endodontic procedures [45]. These observations provide biological context for the anatomical findings, but do not establish the clinical relevance of the observed differences in accessory apical foramina; their influence on treatment outcomes was not evaluated in the present study.

Mechanical instrumentation cannot directly reach lateral canals, apical deltas, or accessory openings [46]. Irrigant delivery, replenishment, contact time, and activation techniques such as passive ultrasonic irrigation are relevant to chemical debridement of these areas [47,48], but their effectiveness was not assessed here. The greater frequency and number of accessory apical foramina observed in mesial systems provide an anatomical rationale for investigating these approaches, without demonstrating the efficacy of any specific protocol. Distal systems exhibited a different anatomical pattern: accessory foramina were less frequent, but when present, they had a larger mean perimeter and greater variability. These anatomical differences do not establish greater clinical risk in either system or the effectiveness of specific approaches to working-length control, prevention of irrigant or filling-material extrusion, or three-dimensional obturation.

These findings generate testable hypotheses for future studies. Controlled experiments could test whether the number and two-dimensional perimeter of accessory apical foramina are associated with irrigant penetration, whether activation techniques modify penetration compared with non-activated irrigation, and whether obturation materials differ in their ability to fill these ramifications. Clinical studies with appropriate assessment of anatomy and control of confounding factors could investigate whether accessory foraminal characteristics are associated with post-treatment apical periodontitis. These hypotheses are not demonstrated by the present anatomical data.

This study has several limitations. First, non-probability stratified sampling was used to balance mesial Vertucci Types I, II, and IV, whereas all distal systems were Type I. The reported frequencies therefore describe the selected sample and should not be interpreted as population-level estimates. Second, the sample was restricted to mandibular second molars from Caucasian individuals aged 55–60 years and extracted for periodontal reasons; generalization to younger individuals, other populations, and teeth extracted for different reasons is consequently limited. Specimens showing apical resorption, marked hypercementosis, fractures, or extraction-related damage within the apical 3 mm were excluded to reduce potential anatomical bias. Teeth with partial external root fusion in the middle or apical third were included only when the mesial and distal canal systems remained anatomically independent. Accordingly, the findings should not be generalized to teeth with completely fused roots or C-shaped canal systems.

The restricted age range may also be relevant to apical morphology. Continued dentin deposition can reduce root canal dimensions with age, whereas cementum apposition and apical remodeling may alter the position, width, and contour of the apical foramen [49,50]. Consequently, the anatomy observed in teeth from individuals aged 55–60 years may not represent that of younger patients. The periodontal indication for extraction introduces an additional potential selection effect: although specimens with visible apical resorption, marked hypercementosis, or extraction-related damage were excluded, subtle changes associated with long-term function, periodontal disease, or the external apical tissues cannot be excluded from an ex vivo morphological assessment. Likewise, documented population-related variation in root and canal anatomy limits extrapolation from this Caucasian sample to other ethnic groups [13]. The direction and magnitude of these possible influences cannot be determined from the present design. Future studies should therefore include probability-based or consecutively assembled samples spanning broader age ranges, multiple populations, and different extraction indications.

The perimeter was measured on a predefined foraminal reference section and therefore represents a two-dimensional boundary rather than the complete three-dimensional morphology of the opening on the external root surface. Perimeter was a secondary endpoint evaluated only in canal systems containing at least one accessory apical foramen. Because accessory foramina were present in both canal systems in some teeth but only in one system in others, the analysis included a mixture of paired and unpaired positive systems. The GEE analysis accounted for dependence between systems from the same tooth through tooth-level clustering and robust variance estimation. Nevertheless, the comparison was conditional on the presence of accessory foramina and does not estimate an unconditional mesial–distal difference across all canal systems. A sensitivity analysis restricted to the 15 teeth with accessory foramina in both systems provided a fully paired comparison within that subset. In addition, the image stack was not reoriented so that each accessory exit was sectioned perpendicular to its local axis. An oblique exit could therefore appear elongated and yield a larger perimeter than the same opening measured orthogonally. Selecting the section on which communication with the external root surface was most clearly delineated, together with verification on adjacent sections and three-dimensional reconstructions, standardized identification but could not eliminate this orientation-dependent effect. Observed perimeter differences may consequently reflect exit orientation as well as opening dimensions, and no clinical cutoff should be inferred from these measurements.

Although the measurements were verified by a second examiner and uncertain findings were resolved by consensus, separate pre-consensus assessments were not retained. Consequently, quantitative interobserver agreement could not be calculated retrospectively, and this should be considered when interpreting the reproducibility of the identification and morphometric assessment of small accessory foramina. In addition, the three-voxel eligibility threshold and visual verification procedures do not eliminate uncertainty associated with partial-volume effects, segmentation, and contour delineation, particularly for openings close to the threshold. These factors may affect both the number of detected accessory foramina and their measured perimeter.

Finally, because of the ex vivo design, the anatomical findings cannot be directly associated with clinical outcomes. Microbial persistence, irrigant penetration, obturation quality, periapical healing, and endodontic treatment outcomes were not evaluated. The potential clinical implications should therefore be regarded as hypotheses based on anatomy, rather than as evidence of specific clinical risks or treatment efficacy.

Despite these limitations, the study’s strengths include high-resolution micro-CT imaging, an anatomically well-defined sample with independent mesial and distal canal systems, and statistical methods that account for the paired structure of the primary data. Stratification by mesial Vertucci type also enabled direct evaluation of the relationship between canal configuration and accessory apical foramina.

## 5. Conclusions

Within the limitations of this ex vivo study, accessory apical foramina were more frequent and more numerous per canal system in mesial than in distal systems of mandibular second molars. Among systems containing at least one accessory apical foramen, distal systems exhibited a greater mean system-level two-dimensional perimeter. No statistically significant association was detected between mesial Vertucci type and either the presence or the number of accessory apical foramina. These findings demonstrate that substantial apical complexity may occur in mandibular second molars with anatomically independent mesial and distal canal systems, including teeth with partial external root fusion, and provide an anatomical basis for investigating the entire apical canal system in relation to cleaning, disinfection, and obturation; the clinical effects of these anatomical features require direct investigation. These frequency estimates apply only to the selected stratified sample and should not be extrapolated as population prevalence values, particularly for younger individuals, other ethnic groups, or teeth extracted for different clinical indications.

## Figures and Tables

**Table 1:** Distribution of mesial canal configurations and presence of accessory apical foramina. In all specimens, the distal root exhibited a Vertucci Type I canal configuration.

Mesial Canal Configuration (Vertucci)	No. of Mesial Systems	Positive Mesial *n*/N (%)	Negative Mesial *n*/N (%)
Type I	21	13/21 (61.9%)	8/21 (38.1%)
Type II	21	15/21 (71.4%)	6/21 (28.6%)
Type IV	22	14/22 (63.6%)	8/22 (36.4%)
Total	64	42/64 (65.6%)	22/64 (34.4%)

**Table 2:** Paired distribution of accessory apical foramina in mesial and distal root canal systems. McNemar exact test: *p* = 0.0003; matched odds ratio = 4.5; exact 95% CI: 1.82–13.33.

Mesial System	Positive Distal	Negative Distal	Total
Positive	15 (Both +)	27 (Mesial only +)	42
Negative	6 (Distal only +)	16 (Both −)	22
Total	21	43	64

Note: + indicates presence of accessory apical foramina; − indicates absence of accessory apical foramina.

**Table 3:** Total and mean number of accessory apical foramina for all root canal systems (Wilcoxon signed-rank test, *p* = 0.0002) and for positive systems only.

Canal System	Positive Systems *n*/N (%)	Total No. of Accessory Foramina	Mean in Positive Systems	Mean per All Systems
Mesial	42/64 (65.6%)	152	3.62	2.38
Distal	21/64 (32.8%)	66	3.14	1.03

**Table 4:** Association of Vertucci canal configuration with the presence and number of accessory apical foramina in mesial systems.

Vertucci Canal Configuration	Total Systems, *n*	Positive Mesial Systems, *n* (%)	Overall *p*-Value
Type I	21	13 (61.9%)	
Type II	21	15 (71.4%)	
Type IV	22	14 (63.6%)	
Total	64	42 (65.6%)	0.786 ^1^; 0.577 ^2^

^1^ Pearson’s chi-square test (χ^2^ = 0.48, df = 2, Cramer’s V = 0.087) comparing the frequency of accessory apical foramina among the three configurations. ^2^ Kruskal–Wallis test (H = 1.10, df = 2) comparing the number of accessory apical foramina among the three configurations.

**Table 5:** Descriptive statistics of the mean two-dimensional perimeter of accessory apical foramina at the canal-system level.

Canal System	Positive Canal Systems, *n*	Mean Perimeter ± SD, mm	Median Perimeter (IQR), mm	Range, mm
Mesial	42	0.172 ± 0.028	0.168 (0.151–0.190)	0.114–0.236
Distal	21	0.432 ± 0.176	0.395 (0.302–0.558)	0.186–0.780

Note: Values represent the mean two-dimensional perimeter calculated at the canal-system level. When multiple accessory apical foramina were present within the same positive canal system, their individual perimeters were averaged, providing one observation per positive system. IQR, interquartile range; SD, standard deviation.

**Table 6:** GEE estimates for mesial–distal comparisons accounting for tooth-level clustering.

Outcome and Comparison	Systems/Teeth	Estimate	95% CI	*p*-Value
Accessory-foramen count Mesial/distal	128/64	Mean count ratio 2.30	1.48–3.58	0.000207
Mean system-level perimeter Distal-mesial	63/48	Mean difference 0.260 mm	0.184–0.337 mm	<0.001

GEE, generalized estimating equations; CI, confidence interval. Count analysis: log link and Poisson working variance, including zero counts. Perimeter analysis: Gaussian identity link, restricted to positive systems, with one mean per system. Both models used canal-system location as the sole predictor, tooth-level clustering, an independence working correlation structure, and the Mancl–DeRouen bias-reduced robust sandwich covariance estimator. Confidence intervals and two-sided *p*-values used normal-approximation Wald inference. The count ratio is mesial/distal; the perimeter difference is distal minus mesial. Neither model adjusted for Vertucci configuration.

## Data Availability

The morphometric measurement data supporting the findings of this study are available from the corresponding author upon reasonable request.
